# Longitudinal Investigation of Public Trust in Institutions Relative to the 2009 H1N1 Pandemic in Switzerland

**DOI:** 10.1371/journal.pone.0049806

**Published:** 2012-11-21

**Authors:** Adrian Bangerter, Franciska Krings, Audrey Mouton, Ingrid Gilles, Eva G. T. Green, Alain Clémence

**Affiliations:** 1 Institute of Work and Organizational Psychology, University of Neuchâtel, Neuchâtel, Switzerland; 2 Department of Organizational Behavior, University of Lausanne, Lausanne, Switzerland; 3 Institute of Social Sciences, University of Lausanne, Lausanne, Switzerland; The University of Hong Kong, Hong Kong

## Abstract

**Background:**

The 2009 H1N1 pandemic left a legacy of mistrust in the public relative to how outbreaks of emerging infectious diseases are managed. To prepare for future outbreaks, it is crucial to explore the phenomenon of public trust in the institutions responsible for managing disease outbreaks. We investigated the evolution of public trust in institutions during and after the 2009 pandemic in Switzerland. We also explored respondents’ perceptions of the prevention campaign and the roles of the government and media.

**Methodology/Principal Findings:**

A two-wave longitudinal survey was mailed to 2,400 members of the Swiss public. Wave 1 was in Spring 2009. Wave 2 was in Spring 2010. Six hundred and two participants responded in both waves. Participants indicated moderate to high levels of trust in medical organizations, the WHO, the Swiss government, the pharmaceutical industry, and the EU. On the other hand, trust in the media was low. Moreover, trust in almost all institutions decreased over time. Participants were satisfied with the amount of information received and indicated having followed official recommendations, but widespread concerns about the vaccine were evident. A large majority of participants agreed the vaccine might have unknown or undesirable side effects. Perceptions of the government’s and the media’s role in handling the outbreak were characterized by a substantial degree of skepticism and mistrust.

**Conclusions/Significance:**

Results show clear patterns of skepticism and mistrust on the part of the public relative to various institutions and their actions. Results underscore the importance of systematically investigating trust of the public relative to epidemics. Moreover, studies investigating the evolution of the public’s memories of the pandemic over the coming years may be important to understand reactions to future pandemics. A systematic research program on trust can inform public health communication campaigns, enabling tailored communication initiatives.

## Introduction

On August 10, 2010, the end of the 2009 H1N1 pandemic was officially declared by the World Health Organization [1]. The timeline of the disease and related consequences are well known. Soon after its initial emergence in Mexico and the USA, H1N1 (swine flu) spread rapidly over the planet, prompting the WHO to declare the pandemic alert phase on June 11, 2009, in effect instituting the first influenza pandemic of the 21st century. Nations worldwide ordered millions of doses of vaccine, many of which arrived after the main waves of infections peaked, in the final months of 2009. Vaccination rates were low [2]. In the aftermath of the vaccination campaigns, many countries were left with substantial quantities of unused vaccine. The epidemiological consequences of the pandemic were much less serious than expected and public health concerns have since shifted from classic issues like containing the disease spread and limiting its impact on human societies to an unprecedented issue that has since emerged, namely a *crisis of public trust* in the national and international institutions involved in managing the disease outbreak [3].

Social science research on trust distinguishes between trust in the *competence* or the *motives* of an agent or an institution [4], [5]. The crisis of public trust involves both aspects. There does seem to be widespread mistrust regarding institutional competence in managing disease outbreaks. An example is the belief that the vaccine has serious side effects that are currently unknown to scientists [6]. Another example is skepticism on the part of the public towards the accuracy of official risk assessments of infectious diseases like H5N1 and H1N1 influenza, as well as the utility or efficacy of institutional action. On the other hand, there also seems to be a widespread mistrust regarding the motives of institutions in managing disease outbreaks. An example of this is the belief that risk has been intentionally exaggerated by powerful interest groups for ulterior motives [7]. Both types of mistrust require urgent attention on the part of researchers.

Public mistrust may have its proximate origins in the 2005–2006 H5N1 outbreak, but potentially hark back to earlier affairs like the counterproductive 1976 swine flu vaccination campaign [8] or even the Tuskegee syphilis experiments that have left a lingering legacy of mistrust in some social groups [9]. It is also possible that the current crisis has been influenced by the related issue of vaccination skepticism that is propagated by vocal activist groups through the media [10] and is gaining credence in the mainstream public [11]. The handling of the pandemic by organizations like the WHO has come under critical scrutiny from political and scientific authorities as well as journalists [12], who have raised accusations of conflict of interest. More generally, perceptions of government “cover-up” in various past food and health scares (e.g., mad cow disease) may also affect trust in the long term. These events may influence public sentiment that non-profit organizations or governments are influenced by the pharmaceutical industry, with some commentators even suggesting that the outbreak was purposely engineered to sell vaccine [13].

The crisis of public trust has important negative effects. People who believe that the swine flu outbreak was exaggerated are less prone to change their behavior to comply with recommended protection measures [14]. Beliefs that the virus is not severe are associated with decreased vaccination intent [15]. And actual vaccination uptake is predicted by prior trust in medical organizations [16]. But perhaps the most far-reaching potential effects of the crisis may emerge in the long term, by detrimentally affecting the public’s reactions to future influenza outbreaks, leading to fewer changes in behavior, reduced compliance and, ultimately, failure of health campaigns. Analyses of other cases of failed health programs suggest that this is a plausible outcome [3].

Understanding public trust relative to emerging influenza outbreaks thus becomes a significant priority, both in itself and in preparation for future disease outbreaks. Unfortunately, little is currently known about this topic. Trust needs to be studied in a more differentiated manner distinguishing between trust in competence and motives as well as whether they lead to different behavioral outcomes. Studies should also ascertain which institutions are trusted and which are not. Moreover, data on the content of public perceptions of how the crisis was handled are needed. Finally, it is necessary to explore how levels of trust fluctuate over time, in other words, longitudinal studies are needed. Current research fails on several of these aspects. Studies of public perceptions are often cross-sectional in nature [17] and hence do not permit tracing dynamic evolutions. Existing longitudinal studies [18] do not explore trust in detail, e.g., by studying trust in different institutions. At the same time, the recent influenza outbreaks are characterized by a high degree of uncertainty which limits the extent to which classic health communication strategies can be applied. For example, recommendations to increase media coverage [19] and thus set an agenda for public concerns may backfire if trust in media reporting is low [20]. Indeed, qualitative data suggests that media coverage of the pandemic was often perceived as exaggerated [21], [7]. But such studies often are not representative. As a result of these issues, the evidence base on which to design future health campaigns is thin – a worrying state of affairs and at the same time a potential opportunity to contribute to the process of rebuilding trust during the current interpandemic period [3].

Our research addresses several of these issues. We conducted a longitudinal study in Switzerland with the goal of tracking the content and evolution of the public’s trust in various institutions involved in managing the pandemic, between its initial outbreak in Spring 2009 and its aftermath a year later in Spring 2010. In order to understand the legacy of the H1N1 pandemic in the public’s eye, we also measured the public’s perceptions in Spring 2010 of (1) the prevention campaign, (2) the role of the government in managing the outbreak and (3) the role of the media in covering the outbreak.

**Figure 1:**
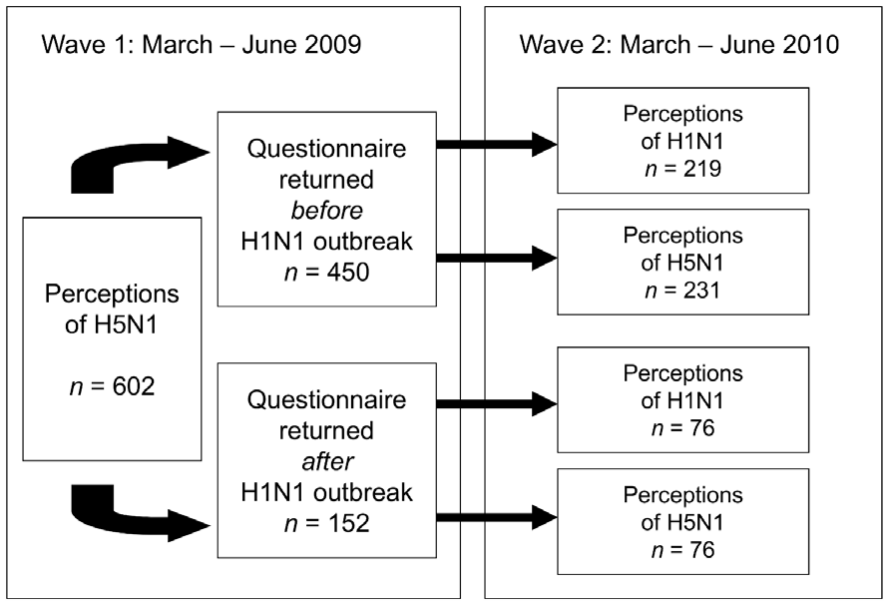
Study Design.

Our study was originally designed as a two-wave longitudinal exploration of public perceptions of avian influenza (H5N1) risk via a survey mailed to potential participants. Wave 1 of data collection was launched in Spring 2009. The 2009 H1N1 outbreak serendipitously occurred during data collection. We seized the opportunity to design Wave 2 of data collection so as to investigate the abovementioned issues about H1N1 influenza. We thus included questions about perceptions of the “swine flu” crisis and its handling in Wave 2. However, to control for possible effects of the H1N1 outbreak during Wave 1 data collection, we marked Wave 1 surveys returned after the beginning of the outbreak (Figure 1).

Given that H5N1 and H1N1 are both strains of influenza and that their respective outbreaks occurred within a few years of each other, the public may exhibit comparable levels of trust in institutions regarding the management of these two diseases. On the other hand, these two diseases differ markedly in certain characteristics, e.g., their mortality rate. It is therefore an open question whether the public’s perceptions of the trustworthiness of institutions differ for the two diseases. Since Wave 1 measured trust in institutions relative to managing H5N1, we split the sample in Wave 2, asking half the participants to evaluate trust in institutions relative to managing H5N1 and half to evaluate trust in institutions relative to managing H1N1 (Figure 1). With this design, then, half the sample evaluates trust relative to H5N1 in Waves 1 and 2, and half the sample evaluates trust relative to H5N1 in Wave 1 and trust relative to H1N1 in Wave 2. This allows us to ascertain with precision whether the public’s trust is different for these two diseases or not.

## Methods

### Ethics Statement

The University of Neuchâtel and the University of Lausanne do not have institutional review boards for psychology or social science research. We thus applied the ethical standards of the Swiss Psychological Society, via its recommended ethics checklist (“Checkliste für die ethische Beurteilung von Psychologischen Forschungsvorhaben”, see www.ssp-sgp.ch/ethik.htm). According to this checklist, our questionnaire avoids any treatment that might have a detrimental effect on the well-being or integrity of participants. Participants were informed about the purpose of the study in a cover letter, and were ensured their data would remain confidential. Agreement to participate by filling out and returning the questionnaire was taken as consent.

### Data Collection

We conducted a longitudinal study in the French-speaking part of Switzerland. The study had two measurement points: March to June 2009 (Wave 1) and March to June 2010 (Wave 2). We employed a quota sampling procedure designed to obtain equal proportions of men and women, equal proportions of three age groups (20–39, 40–65,>65), and equal proportions of people residing in rural or urban areas. Participants’ addresses were selected from a large commercial database. For Wave 1, 2,400 adult participants were sent a questionnaire by mail, together with a pre-paid return envelope, addressed to the university. We followed up on the initial contact with a reminder letter three weeks later. Nine hundred fifty questionnaires were mailed back (response rate = 39.6%). Respondents were contacted again one year later, to participate in Wave 2, using the same procedures as for Wave 1. Six hundred two usable questionnaires were returned (response rate = 63.4%). In both waves, participants received 20 CHF for filling out the questionnaire. As outlined above (Figure 1), questionnaires in Wave 1 focused on perceptions of H5N1. To control for the outbreak of H1N1 during the data collection, questionnaires were coded as having been returned before (*n* = 450) or after the H1N1 outbreak (*n* = 152). In Wave 2, half of the respondents (*n* = 295) filled out a questionnaire on perceptions of H1N1, the other half (*n* = 307) on perceptions of H5N1. The H5N1 questionnaire also featured some items about H1N1 (see *Measures* below). Importantly, we only aggregated the data of Wave 2 across the two main target diseases if preliminary analyses indicated that there were no differences in disease perceptions.

### Measures

In Wave 1, we assessed participants’ gender, subjective health and germ aversion [24] as control variables. In Wave 2, we assessed perceptions of the disease prevention campaign and perceptions of the roles played by the government and the media. Questions were designed to capture key elements of public sentiment as identified in earlier qualitative studies [7]. In both waves, we assessed trust in eight institutions involved in dealing with either H5N1 (Wave 1) or H1N1/H5N1 (Wave 2), thus allowing us to analyze changes in trust. Measures are described in detail below. If not indicated otherwise, participants indicated their responses on 5-point Likert scales using the following anchors: 1 = *do not agree at all*, 2 = *do not agree*, 3 = *agree somewhat*, 4 = *agree*, 5 = *strongly agree*).

#### Subjective health

In Wave 1, subjective health was measured with one item *How is your health in general?* (1 = *very bad*, 2 = *bad*, 3 = *okay*, 4 = *good*, 5 = *very good*; *M* = 4.10, *SD* = 0.73).

#### Germ aversion

Germ aversion is a subscale of the Perceived Vulnerability to Disease scale [24]. Germ aversion captures inter-individual differences with respect to “discomfort in situations that connote an increased likelihood for the transmission of pathogens” (p. 545) [24]. Sample items are *It really bothers me when people sneeze without covering their mouths* or *I prefer to wash my hands pretty soon after shaking someone's hand*. Items were averaged into one scale, with higher values indicating higher levels of germ aversion (α = 0.74, *M* = 2.50, *SD* = 0.64).

#### Trust in institutions

Trust in institutions pertaining to combating the virus was measured in Waves 1 (H5N1) and 2 (both H5N1 and H1N1). Participants’ indicated their level of trust (1 = *no trust at all*, 5 = *a lot of trust*) in eight institutions: the *Swiss government*, *governments of countries where the virus has affected humans*, the *European Union*, the *World Heath Organization*, *medical organizations*, the *agro-food industry*, the *pharmaceutical industry*, and the *media*. Items showed high internal consistency in both waves (Wave 1 α = .83, Wave 2 α = .86), but we analyzed them separately in what follows in order to understand institution-specific trust perceptions.

#### Perceptions of the disease prevention campaign

Respondents indicated their opinions relative to the following four items: *Do you think the vaccine was useful?*, *Did you follow the recommendations of the Federal Office of Public Health (wash hands, sneeze into sleeve…)?*, *Do you think you have received enough information on the part of health authorities about the vaccination procedure?* and *Do you think the vaccine can have unknown/problematic side effects?* Responses were made using a 5-point Likert scale (1 = *No, not at all*, 2 = *No*, 3 = *Neither yes nor no*, 4 = *Yes*, 5 = *Yes, absolutely*). Items showed low internal consistency (α = .39), so we analyzed them separately in what follows, especially in order to differentiate among perceptions of different aspects of the campaign.

#### Perceptions of the role of the government

Participants’ perceptions of the role of the Swiss government in managing the H1N1 outbreak were assessed in Wave 2 (for the H1N1 questionnaire only, *n* = 152), with the following three items: *The government accentuated the crisis in order to exhaust Tamiflu stocks*, *The government omitted or hid certain relevant facts*, *The government put too much pressure on people to encourage them to get vaccinated*. Items showed high internal consistency (α = .80), but we analyzed them separately in what follows, in order to differentiate among perceptions of different aspects of the government’s actions.

#### Perceptions of the role of the media

Participants’ perceptions of the role the media played in covering the current H1N1 outbreak or the earlier H5N1 outbreak were assessed with five items in Wave 2: *The media have exaggerated the risk posed by this disease*, *Information in the media helped avoid an outbreak of cases in Switzerland* (reverse-scored), *The media have omitted or hidden some relevant facts*, *Extensive media reporting was necessary to attract people’s attention to the dangers of the flu* (reverse-scored), and *One cannot trust what one hears in the press about this disease*. Items showed low internal consistency (α = .10), so we analyzed them separately in what follows, especially in order to differentiate among perceptions of different aspects of the media’s actions.

## Results

### Respondent Characteristics

The final longitudinal sample (*n* = 602; 342 women, mean age at Wave 1 46.3 years, *SD* = 15.8 years) was similar to the Swiss population on several key characteristics except for residential area, which we had sampled to equally represent rural and urban populations (Table 1).

**Table 1:** Sample Characteristics Compared to Swiss Population.

		Sample (%)	Population (%)^a^
Sex	Male	43.2	49.2
	Female	56.8	50.8
Age (yrs)	20–39	35.8	33.7
	40–64	46.9	44.9
	>65	14	21.4
Residential Area	Rural	54.6	26
	Urban	45.4	74
Education	Secondary	9.6	13
	Vocational	57	53
	University/college	29.5	34
Monthly Income (CHF)	<3,500	18.3	17
	3,501–9,500	65.4	64.9
	>9,500	15.9	18.1
Vaccination compliance		17.8^b^	14–20^c^

a Population data are from the 2008 census conducted by the Swiss Federal Statistical Office, except for H1N1 vaccination rate. Percentage of population in each age group is computed relative to the population aged 20 and above in order to ensure comparability with the age groups sampled.

b [16].

c http://www.bag.admin.ch/influenza/01120/01134/index.html?lang=fr.

### Trust in Institutions

Correlations between the control variables and the aggregated measure of trust in institutions appear in Table 2. In general, control variables were weakly linked to the trust measure. Women were more trusting than men in Wave 1, but not in Wave 2. Germ aversion correlated with trust in both waves. These results corroborate well-established findings on individual differences in risk perception and trust [22], [23]. Trust at Wave 1 was higher for respondents who returned the surveys after the H1N1 outbreak. The target disease (H5N1 or H1N1) in Wave 2 was not correlated with trust ratings.

**Table 2:** Correlations Between Main Study Variables.

	1.	2.	3.	4.	5.	6.	7.	8.
1. Sex (1 = *female*, 2 = *male*)								
2. Age W1	.011							
3. Subjective Health W1	.047	−.206**						
4. Germ aversion W1	−.145**	.088*	−.285**					
5. W1 survey returned (1 = *before outbreak*, 2 = *after*)	−.005	.003	.068	.068				
6. W2 survey version (1 = *H5N1*, 2 = *H1N1*)	−.009	.003	−.015	.036	.012			
7. Trust in institutions W1	−.122**	−.044	.002	.119*	.095*	.030		
8. Trust in institutions W2	−.046	−.033	−.015	.129**	−.003	−.044	.507**	
9. Increase in trust from W1to W2	.072	.011	−.011	.019	−.099*	−.073	−.436**	.555*

W1: Wave 1. W2: Wave 2.

* *p*<.05.

** *p*<.01.

Most importantly, trust in institutions at Wave 1 did not differ between respondents and nonrespondents at Wave 2. A multivariate analysis of variance (MANOVA) with respondents vs. nonrespondents as between-subjects factor and trust in the eight institutions as dependent variables revealed that overall, trust in institutions did not differ as a function of Wave 2 respondent status, Wilk’s Λ = .99, F(8, 921) = .94, *ns*. The global mean of the scale is 2.99 (*SD* = .66) for Wave 2 nonrespondents versus 2.98 (*SD* = .66) for Wave 2 respondents. Moreover, none of the univariate tests were significant, indicating that this global result also holds for each of the institutions considered individually. Thus, drop-outs between Waves 1 and 2 are not affected by trust in institutions, our focal variable.

Our main purpose was to capture changes in trust in institutions. We thus analyzed differences in trust levels between Waves 1 and 2. We first determined if mean trust levels differed as a function of the main target disease (H1N1 or H5N1) in the Wave 2 questionnaire. A MANOVA with target disease as between-subjects factor (H1N1 or H5N1) and trust in the eight institutions as dependent variables revealed that overall, trust in institutions did not differ as a function of target disease, that is whether questions referred to H5N1 or H1N1 influenza, Wilk’s Λ = .98, *F*(8, 574) = 1.35, *ns*. Hence, for the analyses reported below, we aggregated the data across target diseases.

**Figure 2:**
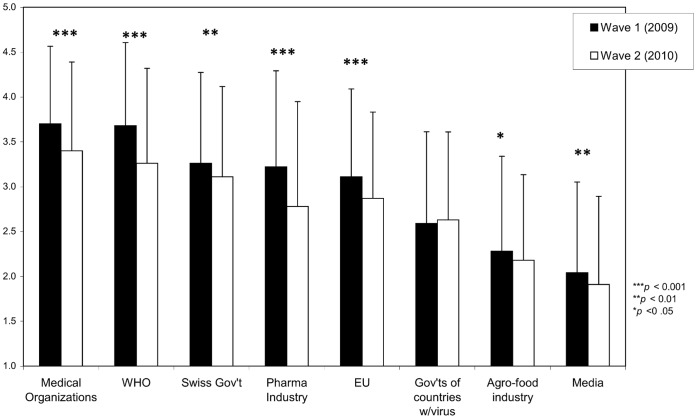
Trust in Institutions, Waves 1 and 2 (error bars indicate one standard deviation).

To analyze changes between Waves 1 and 2, we conducted a MANOVA with wave as within-subjects factor and the eight trust items as dependent variables. Further, we entered participants’ gender, subjective health and level of germ aversion and whether respondents filled out questionnaires of Wave 1 before or after the emergence of H1N1 as covariates into the analysis, to control for their potential impact on levels of trust. Results revealed that overall, trust in institutions decreased significantly from Wave 1 to Wave 2, Wilk’s Λ = .84, *F*(7,561) = 14.73, *p*<.001 (Figure 2). Univariate analyses showed that trust levels decreased for all institutions, all *F*s>5.12, all *p*s<.05, except for trust in governments of countries that are affected by the disease, *F*(1,593) = 0.79, *ns*. Mean levels of trust were highest for medical organizations, followed by the WHO and the Swiss government. Interestingly, trust in the media was lowest. Absolute mean-level decreases in trust from Wave 1 to Wave 2 were largest for the World Health Organization, the pharmaceutical industry, and medical organizations (Figure 2).

### Perceptions of the Disease Prevention Campaign

**Figure 3:**
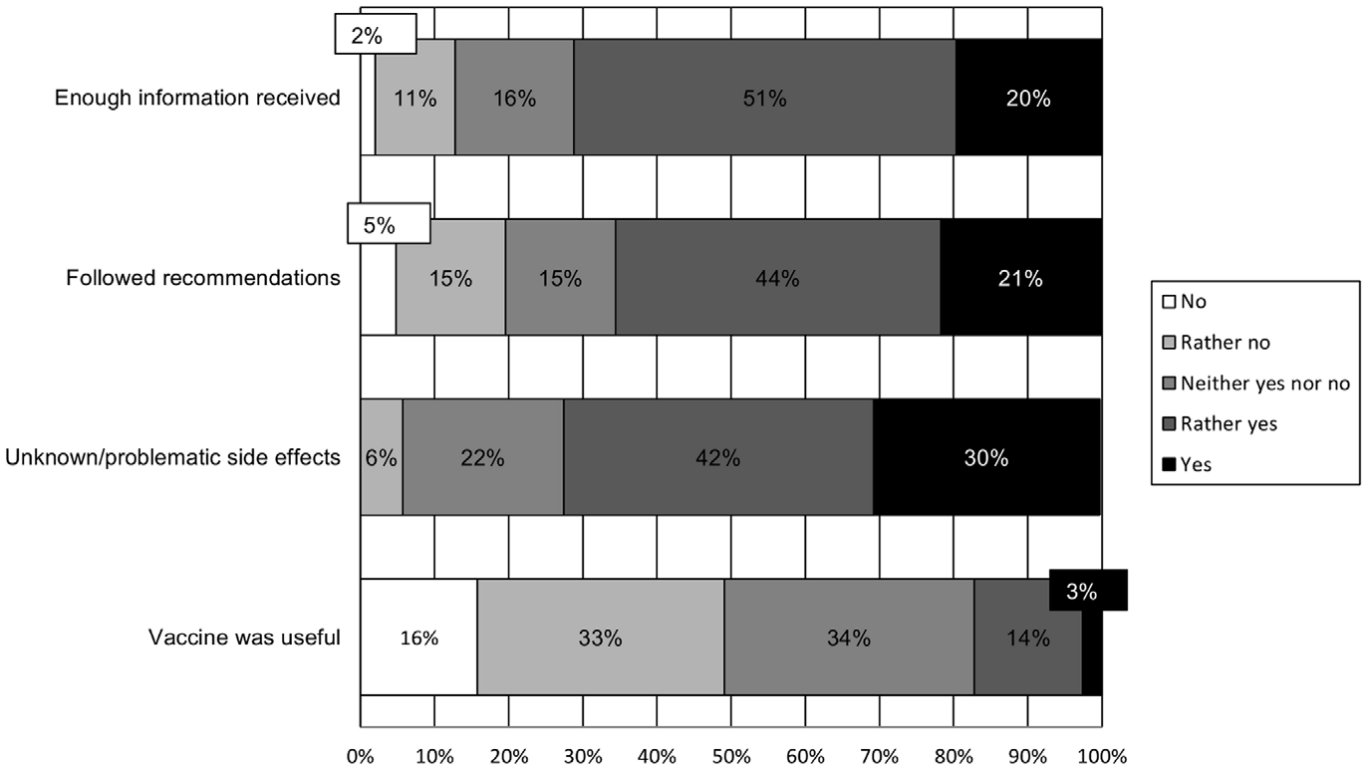
Perceptions of the Disease Prevention Campaign, Wave 2.

We first tested if the role of the media was perceived differently depending on the target disease in the questionnaire. Results of a MANOVA with the four disease prevention campaign items as dependent variables and target disease (H1N1 or H5N1) as between-subjects independent variable showed that this was not the case, Wilk’s Λ = 1.0, *F*(4, 591) = .31, *ns*, allowing us to aggregate the data across questionnaires. Descriptive data are shown in Figure 3. The majority of respondents felt that they had received enough information from health authorities on the vaccination procedure (71% responding *rather yes* or *yes*) and indicated having followed the recommendations of the Federal Office of Public Health (65% responding *rather yes* or *yes*). Nevertheless, 72% of the respondents thought that the vaccine could have unknown or problematic side effects (responding *rather yes* or *yes*). And 49% did not consider the vaccination useful (responding *rather no* or *no*).

### Perceptions of the Role of the Government

**Figure 4:**
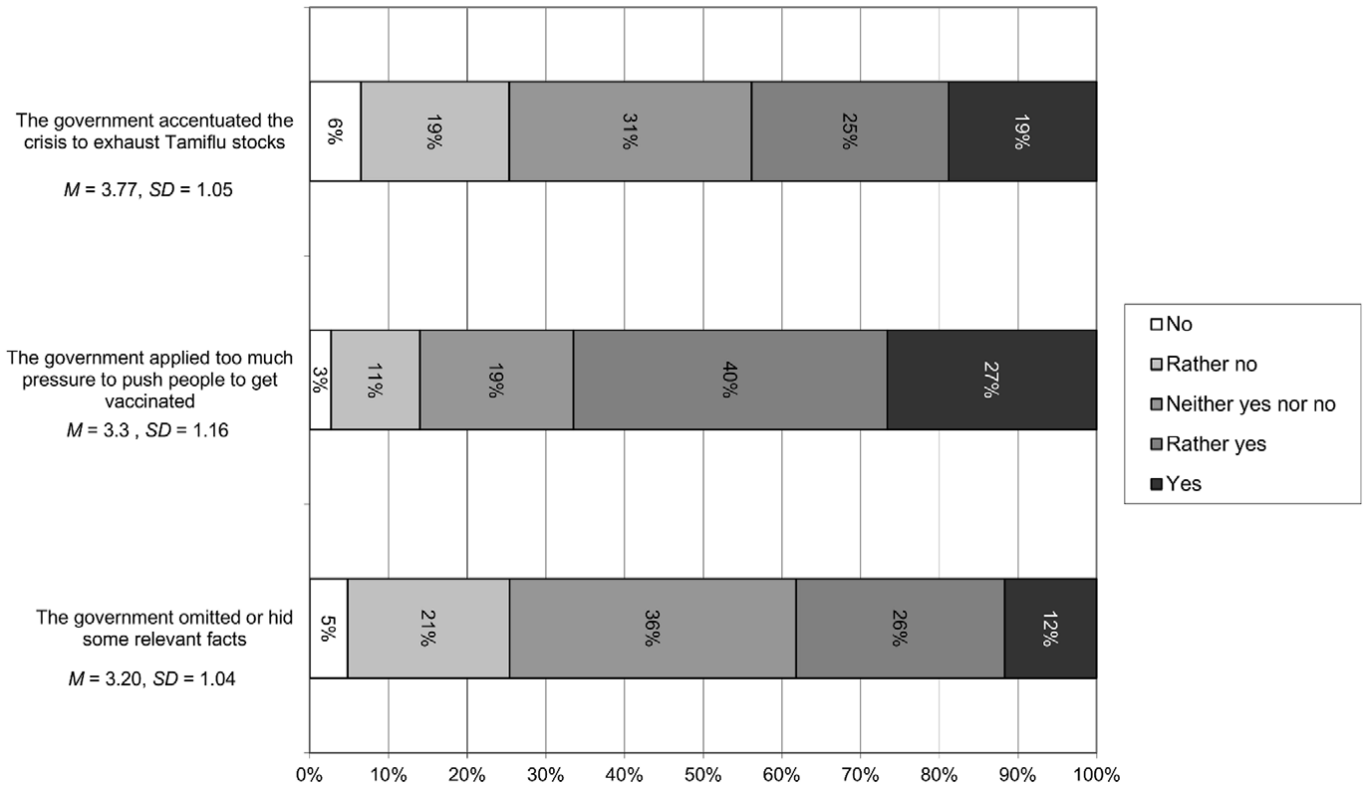
Perceptions of the Role of the Government, Wave 2.

Descriptive data are shown in Figure 4. Forty-four percent of respondents believed that the Swiss government had accentuated the crisis in order to exhaust Tamiflu stocks (responding *rather yes* or *yes*) and 67% (responding *rather yes* or *yes*) felt that the government had put too much pressure on people to get vaccinated. Also, 38% (responding *rather yes* or *yes*) believed that the government had omitted or hid some relevant facts.

### Perceptions of the Role of the Media

We first tested if the role of the media was perceived differently depending on the target disease in the questionnaire. Results of a MANOVA with the four media perception items as dependent variables and target disease (H1N1 or H5N1) as between-subjects independent variable showed that this was not the case, Wilk’s Λ = .98, *F*(4, 597) = 2.29, *ns*, allowing us to aggregate the data across questionnaires.

**Figure 5:**
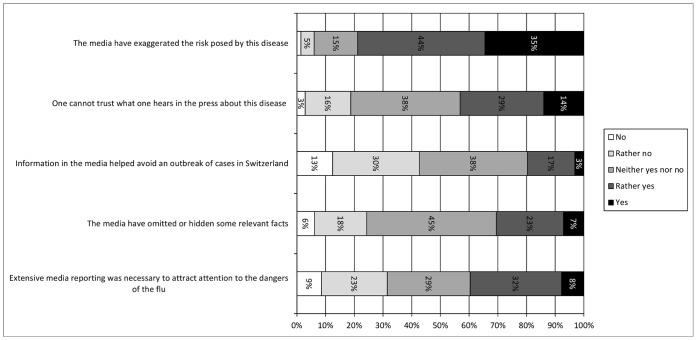
Perceptions of the Role of the Media, Wave 2.

Descriptive data are shown in Figure 5. The majority of respondents (79% responding *rather yes* or *yes*) believed that the media had exaggerated the risk of H1N1 or H5N1. Almost half (43% responding *rather yes* or *yes*) felt that one couldn’t trust what one heard in the press or disagreed that information in the media helped avoid an outbreak of cases (43% responding *rather no* or *no*). A sizeable minority (30% responding *rather yes* or *yes*) believed that the media had omitted or hid some relevant facts. However, respondents also acknowledged the media’s role in drawing people’s attention to the disease’s dangers (40% responding *rather yes* or *yes*).

## Discussion

Our results confirm that public trust is a complex and multifaceted issue. Participants indicated moderate to high levels of trust in some institutions, including medical organizations, the WHO, the Swiss government, the pharmaceutical industry, and the EU. On the other hand, their trust in others, e.g., in the media, was low. Moreover, trust in almost all institutions decreased between the beginning of the outbreak and a year later. The magnitude of the decrease was particularly high for two institutions benefiting from a relatively high level of initial trust, i.e., the WHO and the pharmaceutical industry. This joint decrease may be due to the allegations of conflict of interest linking these two actors, allegations that had been covered in the media at the beginning of 2010, just prior to Wave 2 [12]. Trust in medical organizations decreased but remained the highest among the institutions we investigated, corroborating other studies suggesting that trust in medical professionals is relatively intact [25], [7].

Perceptions of the prevention campaign were also multifaceted. While participants generally were satisfied with the amount of information received and indicated having followed miscellaneous recommendations of the Federal Office of Public Health, widespread concerns about the vaccine were evident. In particular, a large majority of participants agreed the vaccine might have unknown or undesirable side effects, echoing highly publicized but not always accurate popular sentiment about vaccination [10]. Perceptions of the government’s role were characterized by a substantial degree of mistrust in competence and even in motives, as evidenced by the relatively high proportions of participants endorsing the statements that the government may have applied too much pressure to push the vaccination campaign or even acted for ulterior motives (e.g., hiding relevant facts, accentuating the crisis to get rid of excess vaccine stocks). A clear undercurrent of conspiracy thinking is present in these response patterns, corroborating other, qualitative studies [21], [7]. The pattern of mistrust also holds for perceptions of the media, who are largely perceived as crying wolf and also suspected of ulterior motives.

Our study suffers from some limitations. First, given the opportunistic nature of our study, we have investigated H5N1 and H1N1 together. These diseases are different in some of their epidemiological characteristics (e.g., mortality). Nonetheless, they are similar in terms of the reactions they elicit in laypersons, and are indistinguishable from one another in the terms of our analyses. Many studies have shown that public reactions to various emerging infectious diseases follow similar and predictable patterns [26]. Second, we have no guarantee that our sample is representative of the Swiss population. Our results might be biased in the sense that potential respondents who are especially mistrustful of public and private institutions may have chosen not to participate. Our results may thus underestimate the degree to which mistrust is prevalent. Third, even if our results are not biased, we cannot guarantee that they will generalize to other national contexts. Inevitably, the level of trust or mistrust and their targets will depend on the particulars of each context, including the orchestration of the vaccination campaign, the characteristics of local mass media, and other local factors. For example, the Swiss context is characterized by the presence of several large pharmaceutical companies, which may affect the public’s attitudes. For this reason, it is important to conduct similar studies as ours in other national contexts.

Despite these limitations, results show clear patterns of mistrust on the part of the public relative to various institutions and their actions. These results have been tangentially discussed in other national contexts as well [21], underscoring the importance of rebuilding trust, especially in the current interpandemic period and in view of the certain emergence of similar future pandemics [3]. However, such a process should also be guided by a systematic research program, which is currently lacking. The study of trust in the epidemiological context has been to date largely an incidental concern in existing studies. Three aspects need to be remedied in our view. First, future research should draw on the sophisticated conceptual models of trust that exist in the social sciences. A distinction should be made between trust in competence and trust in motives [4], [5]. Laypersons may well differ in their attitudes relative to these particular dimensions for any given institution. Second, research needs to undertake more differentiated studies of the targets of trust, i.e., the different institutions, either private or public, or local, national or international that collaborate in containing and managing a pandemic, and in communicating with the public. Many laypersons probably have undifferentiated views of the institutional actors involved in such situations, and this state of affairs may facilitate perceptions of collusion or conspiracy between actors. A particularly important target for future studies might be trust in front-line health professionals like primary care physicians or nurses. Indeed, laypersons often turn to such professionals for advice on interpreting vaccination recommendations [15]. If these professionals’ own vaccine uptake is low, then their advice may influence laypersons to not get vaccinated [27]. Results from studies focusing on specific targets of trust can inform public health communication campaigns, such that more tailored and participative communication initiatives can be undertaken, beyond purely “top-down” communication [28]. Third, longitudinal research is needed to investigate dynamic aspects of trust. While is it obviously not possible any more to design prospective studies comparing the acute aspects of public reactions during the pandemic to the current aftermath, archival data, such as internet postings and online commentary [21] may be amenable to analysis of longitudinal patterns. Moreover, studies investigating the evolution of the public’s memories of the pandemic over the coming years may be important to understand trust-related reactions to future pandemics.
